# NRSF/REST-Mediated Epigenomic Regulation in the Heart: Transcriptional Control of Natriuretic Peptides and Beyond

**DOI:** 10.3390/biology11081197

**Published:** 2022-08-10

**Authors:** Hideaki Inazumi, Koichiro Kuwahara

**Affiliations:** 1 Department of Cardiovascular Medicine, Graduate School of Medicine, Kyoto University, 54 Shogoin-kawahara-cho, Sakyo-ku, Kyoto 606-8507, Japan; 2 Department of Cardiovascular Medicine, School of Medicine, Shinshu University, 3-1-1 Asahi, Nagano 390-8621, Japan

**Keywords:** heart failure, natriuretic peptides, transcription factors, epigenetics, epigenome

## Abstract

**Simple Summary:**

Reactivation of the fetal cardiac gene program, such as those encoding atrial and brain natriuretic peptides (ANP and BNP, respectively), is a characteristic feature of failing hearts. We previously revealed that a transcriptional repressor, neuron-restrictive silencer factor (NRSF), also called repressor element-1-silencing transcription factor (REST), plays a crucial role in the transcriptional control of ANP, BNP and other fetal cardiac genes through collaboration with various other transcription factors to maintain physiological cardiac function and electrical stability. Increased production of ANP and BNP prevents the progression of heart failure, but reactivation of Gα_o_ and fetal-type cardiac ion channels (T-type Ca^2+^ and HCN channels) leads to deteriorated cardiac function and lethal arrhythmias observed in mice with disturbed NRSF function. Epigenetic regulators with which NRSF forms a complex modify histone acetylation and methylation, thereby participating in NRSF-mediated transcriptional regulation. Further comprehensive studies will lead to clarification of the molecular mechanisms underlying the development of cardiac dysfunction and heart failure.

**Abstract:**

Reactivation of fetal cardiac genes, including those encoding atrial natriuretic peptide (ANP) and brain natriuretic peptide (BNP), is a key feature of pathological cardiac remodeling and heart failure. Intensive studies on the regulation of ANP and BNP have revealed the involvement of numerous transcriptional factors in the regulation of the fetal cardiac gene program. Among these, we identified that a transcriptional repressor, neuron-restrictive silencer factor (NRSF), also named repressor element-1-silencing transcription factor (REST), which was initially detected as a transcriptional repressor of neuron-specific genes in non-neuronal cells, plays a pivotal role in the transcriptional regulation of ANP, BNP and other fetal cardiac genes. Here we review the transcriptional regulation of ANP and BNP gene expression and the role of the NRSF repressor complex in the regulation of cardiac gene expression and the maintenance of cardiac homeostasis.

## 1. Introduction

Heart failure remains a leading cause of morbidity and mortality worldwide [1,2]. Under conditions of pathological stress or tissue injury, cardiac myocytes develop hypertrophy as an initially adaptive response. The sustained stress, however, makes the hypertrophy pathological and provokes pathological cardiac remodeling, which finally causes heart failure. Increased cell size, increased protein synthesis and sarcomere assembly, and altered gene expression are characteristic features of cardiomyocyte hypertrophy. Among the cardiac gene reprogramming during the pathological cardiac remodeling, there is reactivation of “fetal” cardiac genes; such genes encoding atrial natriuretic peptide (ANP) and brain natriuretic peptide (BNP), fetal isoforms of contractile proteins (skeletal α-actin and β-myosin heavy chain), fetal-type cardiac ion channels and some smooth muscle proteins (smooth muscle α-actin and smooth muscle 22α) are robust markers of cardiac hypertrophy and pathological cardiac remodeling [3]. Fetal ventricles abundantly express these genes, but as the heart matures, the expression is decreased. Reactivation of the fetal gene program plays an important role in the molecular processes underlying pathological cardiac remodeling that alters cardiac structure and function besides acting as a marker of pathological conditions [3]. Indeed, the expression of ANP and BNP in cardiomyocytes is remarkably increased in failing hearts and the elevation of their plasma levels is widely recognized as a prognostic marker of disease severity [4,5].

As a means of better understanding the underlying molecular mechanisms of heart failure, much effort has been made to elucidate the mechanisms regulating expression of fetal cardiac genes, including *NPPA* and *NPPB*, the genes respectively encoding ANP and BNP. It is now known, for example, that fetal cardiac gene programs are regulated by numerous transcriptional factors [6]. Among these, we have been studying the role played by the transcriptional repressor neuron-restrictive silencer factor (NRSF), also named repressor element-1-silencing transcription factor (REST). Normally, NRSF negatively regulates the transcription of *NPPA* and *NPPB* [7,8]. NRSF also represses expression of other fetal cardiac genes, including *HCN2* and *−4* and *CACNA1H*, which respectively encode hyperpolarization-activated cyclic nucleotide-gated (HCN) channels and the T-type Ca^2+^ channel. By forming a complex with several corepressors, NRSF is involved in various molecular pathways that affect the expression of fetal cardiac genes. Consistent with these findings, cardiac-specific inactivation of NRSF by overexpression of a dominant-negative mutant of NRSF (dnNRSF), driven by the cardiac-specific α-MHC promoter (dnNRSF-Tg mice) or cardiac-specific NRSF knockout (NRSF cKO), induces reactivation of fetal cardiac gene expression in the ventricle. Moreover, both dnNRSF-Tg and NRSF cKO mice show deteriorated cardiac function with dilation of the left ventricle and sudden death due to ventricular arrhythmias, indicating that NRSF maintains cardiac integrity by governing the cardiac gene program [9,10]. In this review article, we describe the transcriptional regulation of ANP and BNP mediated through NRSF and other transcriptional factors and the role played by the NRSF repressor complex in maintaining normal cardiac homeostasis.

## 2. Transcriptional Regulation of ANP and BNP in the Heart

ANP and BNP are cardiac-derived peptide hormones that comprise the natriuretic peptide system [11,12]. These two peptides have similar structures, but are synthesized at different sites. ANP is usually synthesized in the atria, while BNP is synthesized mainly in the ventricles [12]. They are cardiac differentiation markers, and their expression is tightly regulated in a spatiotemporal manner during cardiac development. In fact, the analysis of ANP and BNP promoters and their activity made a tremendous contribution to our current understanding of the transcriptional regulation during cardiac development. As for the postnatal period, the expression of ANP and BNP is elevated in the ventricle in various pathological states of the heart, and plasma concentrations of ANP and BNP are remarkably increased in heart failure patients [4]. In fact, plasma concentrations of ANP and BNP are measured clinically to diagnose heart failure, assess prognosis and determine therapeutic strategies [13]. Therefore, the molecular pathways underlying the reactivation of *NPPA* and *NPPB* are thought to be closely related to adaptive or maladaptive signaling pathways evoked by pathological stimuli in the heart. Because of the important role played by ANP and BNP in cardiac physiology and pathology, many studies have been carried out to investigate the molecular mechanisms involved in the regulation of *NPPA* and *NPPB* during cardiac development and disease progression. The regulation of *NPPA* during embryonic development was one of the subjects of early in situ gene expression studies [14]. Those studies showed that *NPPA* expression in the embryonic mouse heart is finely controlled; it is strongly expressed in the atria, while ventricular expression is restricted to the outer “working” myocardium of the left and right ventricles. Later, during embryonic development, strong *NPPA* expression is maintained in the atria, while its ventricular expression becomes limited to the trabeculae. Thus, *NPPA* shows a dynamic expression pattern during embryonic development that is highly restricted to the heart. Research into molecular mechanisms underlying the atrial-specific expression of *NPPA* has promoted the search for cardiac transcription factors, and the ANP promoter has been extensively analyzed by using molecular biological methods.

The proximal part of the 5′ flanking region (5′-FR) of *NPPA* has been shown to be sufficient to recapitulate the spatiotemporal expression of the endogenous gene and to contain sequences crucial for the regulation of *NPPA* expression [15,16]. Expression of a reporter gene driven by the *NPPA* proximal 5′-FR from atrial or ventricular cardiac myocytes obtained at various developmental stages revealed the regions that mediate the proper spatial and temporal expression of the ANP promoter [17,18]. In regard to that point, the *NPPA* proximal 5′-FR contains three T-box binding elements (TBE), two GATA sites, two CArG boxes, two NK-homeobox binding elements (NKE), an A/T-rich element and a phenylephrine-responsive element (PERE), to which the transcriptional factors Tbx5, GATA4/6, SRF, NKX2.5, MEF2C and Zfp260 have all been shown to bind [19,20] and to contribute singly or cooperatively to the basal and inducible activation of ANP promoter in cardiac myocytes [20,21,22,23,24,25,26]. Furthermore, those sequences have been highly conserved in evolutionarily diverse organisms [27]. However, there are some differences in the expression pattern between the proximal 5′-FR of *NPPA* and the intact endogenous *NPPA*, suggesting that regions outside of the proximal 5′-FR region also have a regulatory capacity [19,28]. Investigation of the *NPPA* locus in mouse disclosed that distal regulatory elements are also necessary for fine-tuned regulation of *NPPA* expression during embryonic development [28]. It is reported that the neuron-restrictive silencer element (NRSE), glucocorticoid responsive element (GRE) and hypoxia-response element (HRE), all of which are located outside the proximal promoter, are also involved in the induction of *NPPA* transcription [29,30].

Similarly, the proximal region of the human BNP promoter is sufficient to mediate ventricle-specific *NPPB* expression [31,32]. *NPPB* contains an AT-rich region in its 3′-untranslated region (UTR), which makes the gene unstable, indicating that BNP expression is also regulated post-transcriptionally [33]. Indeed, *NPPB* has a shorter half-life than *NPPA*. Moreover, the region extending from −127 to −40 of the human *NPPB* 5′-FR is found to be necessary for cardiac-specific expression by deletion analysis [32]. This human BNP promoter proximal region contains potential GATA, CArG, AP-1/CRE-like and M-CAT elements, which are conserved among humans, mice and rats [32,34,35,36]. All of these elements have been shown to control the cardiac selectivity of gene expression [35,36,37,38,39,40] and to mediate both basal and inducible expression of *NPPB* [35,36,39,40,41]. Other sites in the distal regions of human *NPPB* 5′-FR, such as NRSE, NF-AT binding sites, SSREs and TRE, have also been found to play important roles in the inducible activation of the human BNP promoter [42,43,44,45].

*NPPA* is located within an insulated chromatin structure called the topologically associating domain (TAD), and *NPPB* and several other genes are located in its vicinity. As TADs generally create a local gene-regulatory environment, it is likely that the specific elements that regulate *NPPA* are situated within this TAD. Further investigation of the *NPPB*-*NPPA* genomic cluster in mice unraveled the specific elements required for cardiac expression of the two genes and demonstrated that physical contact between the genes and their regulatory elements was likely an important component of their co-regulatory expression [46]. For instance, the BNP promoter was shown to be required for stress-induced expression of *NPPA* [46].

Pathological stress including mechanical stress on the heart increases ANP and BNP expression and secretion in both ventricular and atrial myocytes [47,48,49]. During that process, a variety of neurohumoral factors, cytokines and growth factors are induced and cooperatively affect ANP and BNP expression by mediation through various signaling pathways [50]. We previously identified that Rho- and actin-treadmill-dependent nuclear accumulation of myocardin-related transcription factor A (MRTF-A), a coactivator of SRF, contributes to the transduction of mechanical stress to the transcriptional activation of *Nppb* via SRF-responsive element [36].

## 3. NRSF Is a Transcriptional Repressor of Fetal Cardiac Genes, including *NPPA* and *NPPB*

The neuron-restrictive silencer element (NRSE), which is also named as repressor element 1 (RE-1), has been identified as a negatively acting DNA regulatory element that represses neuronal gene expression in non-neuronal cells and in undifferentiated neuronal cells [51,52]. NRSF, also known as REST, has been identified as a protein that binds to NRSE [53]. NRSF is one of the zinc finger transcriptional factor family members and is globally expressed in most non-neuronal tissues, including the heart [52]. Subsequently, it was shown that NRSF also suppresses non-neuronal genes containing NRSE, implying that NRSF is a transcriptional regulator of both neuronal and non-neuronal genes containing NRSE in non-neuronal tissues [54]. As described above, the activity of the 5′-FR of human *NPPA* is strongly suppressed by a fragment containing the 3′-UTR of the gene. The 3′-UTR of *NPPA* contains a conserved sequence similar to NRSE. When the NRSE-like sequence is mutated in the human *NPPA* 3′-UTR, the repressor activity is completely lost. Mutant NRSE also reduces the response of ANP promoter activity to endothelin-1 through the constitutive activation in cultured ventricular myocytes. This indicates that the hypertrophic stimulus-inducible ANP expression in cardiac myocytes is likely attributed to attenuation of NRSE-mediated repression, at least in part [7].

We also found that the 5′-FR of *NPPB* contains a cis-acting negative regulatory element, which has 90% homology to the consensus sequence of NRSE. This element, called the fibronectin-inducible element, is well conserved among species and mediates the increase in BNP promoter activity by fibronectin in cardiomyocytes. We demonstrated that the element binds NRSF, and its mutation significantly increases human BNP promoter activity. These results indicate that NRSF represses transcription of *Nppa* as well as *Nppb* [8]. Indeed, infection of recombinant adenovirus expressing dnNRSF results in an increase of both *Nppa* and *Nppb* expression in cultured ventricular myocytes [9]. Both *Nppa* and *Nppb* expression in ventricles of dnNRSF-Tg are also increased. Adenovirus-mediated expression of dnNRSF in cardiomyocytes prevents hypertrophic stimulation-induced increases in ANP and BNP expression. Furthermore, pressure-load-induced increases in ANP and BNP expression, which are normally seen in wild-type mice, are markedly suppressed in the ventricles of dnNRSF-Tg mice. On the other hand, forced recruitment of NRSF to the ANP promoter in ventricular myocytes represses basal promoter activity, but enhances inducible activity in response to endothelin-1 [7]. Collectively, removal of NRSF-mediated repression likely contributes to the inducible ANP and BNP expression in response to pathological stimuli.

**Figure 1:**
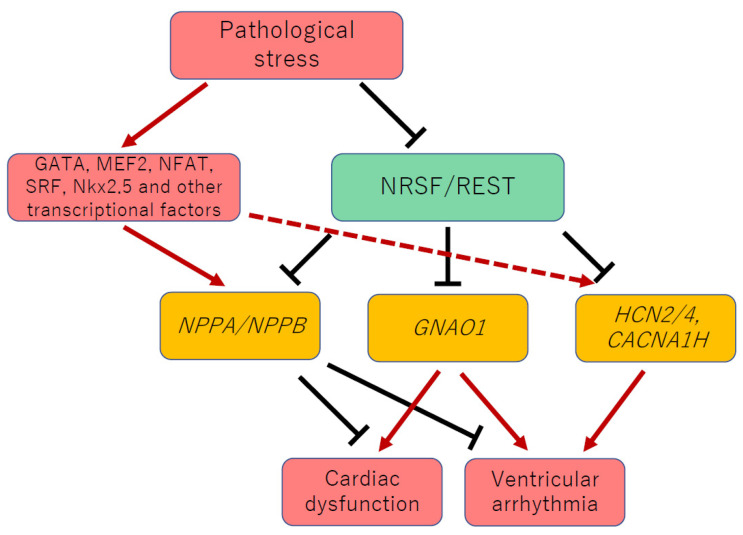
NRSF maintains normal cardiac function and electrical stability by regulating the cardiac gene program with other transcription factors. Whereas ANP and BNP protect against heart failure development, reactivation of Gαo and fetal-type cardiac ion channels, such as T-type Ca^2+^ and HCN channels, leads to cardiac dysfunction and ventricular arrhythmia in dnNRSF-Tg mice and NRSF cKO mice. HCN, hyperpolarization-activated cyclic nucleotide; NFAT, nuclear factor of T cells; MEF2, myocytes enhancer factor 2; SRF, serum response factor; NRSF, neuron-restrictive silencer factor; REST, repressor element-1 silencing factor.

The skeletal α-actin gene is expressed in the fetal ventricle, but its expression level in the ventricle declines after birth and is reactivated only when exposed to pathological stress [9]. 3′-UTR of the skeletal α-actin gene also contains NRSE [55]. This implies that NRSF participates in the maintenance of normal cardiac structure and function by regulating the expression of multiple fetal cardiac genes (Figure 1). As well as *NPPA* and *NPPB*, skeletal α-actin gene expression is significantly elevated in the ventricle of dnNRSF-Tg mice. Intriguingly, dnNRSF-Tg mice exhibit reduced cardiac contractility with left ventricular dilatation, ventricular arrhythmias and early lethality. These indicate that NRSF plays an essential role in maintaining normal myocardial integrity through regulation of the cardiac gene program. Moreover, genetic deletion of guanylyl cyclase-A (GC-A), a common receptor for ANP and BNP, by crossing with GC-A knock out mice, exacerbates the pathological cardiac remodeling observed in dnNRSF-Tg mice, demonstrating that the cardiac dysfunction and lethal arrhythmia observed in dnNRSF-Tg mice are not caused by the increased expression of ANP or BNP [56]. Taking the involvement of NRSF in the reactivation of the fetal cardiac gene program into account, further investigation of the molecular mechanisms by which dnNRSF-Tg mice exhibit cardiac dysfunction and ventricular arrhythmias may bring about the discovery of novel molecular mechanisms underlying the progression to pathological cardiac remodeling and heart failure.

## 4. NRSF Regulates Fetal Cardiac Ion Channels and Maintains Electrical Stability in the Heart

Electrical instability caused by alterations in ion channel activity is likely responsible for the malignant ventricular arrhythmias and sudden arrhythmic death observed in dnNRSF-Tg mice [57]. It has been demonstrated that two types of fetal cardiac ion channel, T-type Ca^2+^ and HCN channels, are potentially responsible for the increased incidence of arrhythmias in dnNRSF-Tg mice (Figure 1). T-type Ca^2+^ channel is one of the well-studied ion channels in fetal myocardium [58]. Voltage-gated Ca^2+^ channels are the main sources of Ca^2+^ influx in excitable cells and are classified into several types: L-(long-lasting), T-(transient), N-(neuronal), P/Q-(Purkinje) and R-(residual-drug-resistant). Among these types of Ca^2+^ channels, cardiac myocytes express only L- and T-type channels. In mature cardiomyocytes, L-type Ca^2+^ channels are the major subtype and play an important role in excitation-contraction coupling [59]. Conversely, T-type Ca^2+^ channels are abundantly expressed in the embryonic ventricle, but their ventricular expression is suppressed during the maturation of the heart, so that their expression is restricted to the conduction system in the adult ventricle [60,61]. However, they are reactivated in hypertrophied and failing ventricles [62]. There are two α1 subunits of T-type Ca^2+^ channel expressed in the heart, α1G (*CACNA1G*) and α1H (*CACNA1H*) [58]. *CACNA1H* contains an NRSE-like sequence within its first intron. This NRSE-like sequence has 93% homology to the NRSE consensus sequence and is well preserved among different mammalian species, including humans. NRSF binds to that sequence, which indicates NRSF-mediated negative regulation of *CACNA1H* expression in cardiac ventricular myocytes [9]. Consistent with this notion, *CACNA1H* expression and T-type Ca^2+^ currents are increased in ventricles of dnNRSF-Tg mice. This suggests that the NRSF-NRSE system is involved in the increased cardiac expression of T-type Ca^2+^ channels observed under pathological conditions. Pharmacological inhibition of T-type Ca^2+^ currents by efonidipine or R(−)-isomer efonidipine significantly prolongs lifespan among dnNRSF-Tg mice and mice with acute myocardial infarction by suppressing electrical abnormalities in ventricular myocytes and consequently ventricular arrhythmias. Although further investigation is necessary, this suggests inhibition of T-type Ca^2+^ channels could be a clinically useful approach to prevent malignant arrhythmias in heart failure patients [63].

The HCN ion channel family (HCN1-4) carries the *I*_f_ current in the heart [64,65]. In the adult mammalian heart, HCN channels are predominantly expressed in the conduction system, especially in the sinus node, where HCN4 controls cardiac rhythmicity as the major isoform [65]. In ventricular myocytes, HCN2 is expressed as the major isoform, though expression level of HCN channels in the healthy adult ventricular myocardium is generally much lower than that in the conduction system. Indeed, *I*_f_ currents are rarely detectable in normal ventricular myocytes [65].

During the developmental process, HCN channels are highly expressed in the ventricular myocardium of the fetus, but their expression gradually decreases after birth and becomes almost exclusively restricted to the conduction system in the adult heart [66]. However, HCN channels, especially HCN2 and HCN4, are re-induced in hypertrophied and failing hearts in both rodents and humans, which leads to an increase in I_f_ currents [67,68]. The transcriptional activator MEF-2 activates HCN4 promoter activity in cardiomyocytes through its binding sequence located in the first intron of the HCN4 gene [69]. Increased HCN2 and HCN4 expression are also observed in dnNRSF-Tg hearts. *HCN4* contains a conserved NRSE-like sequence within its first intron [9]. In neonatal rat ventricular myocytes, a part of the first intron of HCN4 gene containing NRSE-like sequence significantly suppresses the activity of HCN4 promoter in an NRSF-dependent fashion [70]. In addition, during cardiac development, the NRSF expression profile was inversely correlated with the HCN4 expression profile, suggesting that NRSF regulates HCN expression in the developmental stage. Moreover, the NRSE-like sequence seems to be important for the reactivation of HCN4 induced by hypertrophic stimuli [9,70]. Thus, NRSF appears to regulate both basal and inducible expression of the HCN2 and HCN4 genes in ventricular myocytes.

To clarify the role of reactivated HCN channels in the increased arrythmicity associated with heart failure, dnNRSF-Tg mice were treated with ivabradine, a specific HCN channel blocker [71]. Ivabradine given orally at doses as low as 7 mg/kg/day significantly prolonged the life span and reduced incidences of malignant arrhythmias without affecting heart rate and cardiac function or structure. Likewise, in ventricular myocytes isolated from dnNRSF-Tg mice, ivabradine suppressed ventricular arrhythmias by inhibiting pathologically increased automaticity. Reciprocally, cardiac-specific overexpression of HCN2 channels increases susceptibility to arrhythmias induced by β-adrenergic stimulation in mice. These results indicate that increased HCN channel expression in dnNRSF-Tg ventricles likely contributes to increased arrhythmogenicity. From a clinical point of view, it is noteworthy that HCN2 and HCN4 gene expression is increased in hypertrophied and failing human ventricles. The SHIFT study revealed that the beneficial effect of ivabradine in heart failure patients is correlated with heart rate reduction, although there is still a possibility that ivabradine may exert cardioprotective effects independent of heart rates [72,73].

## 5. NRSF Maintains Ca^2+^ Homeostasis and Systolic Function in the Heart

Progressive impairment in cardiac function and dilatation of cardiac chambers are observed in both dnNRSF-Tg mice and NRSF cKO mice; this indicates the essential role played by NRSF in maintaining cardiac integrity. Analysis comparing the gene expression profiles of ventricles between dnNRSF-Tg mice and NRSF cKO mice showed that gene expression of *Gnao1*, which encodes Gα_o_, was increased in both types of mouse, while expression of *Gnai2*, which encodes Gα_i2_, was unchanged.

Gα_o_ and Gα_i2_ belong to the Gα_i/o_ family of heterotrimeric GTP-binding proteins (G proteins). Heterotrimeric G proteins fall into four major families: Gα_s_, Gα_i/o_, Gα_q_ and Gα_12/13_ [74,75,76]. Though Gα_i/o_ activity is reported to be increased in the failing human heart [77,78], its pathophysiological role in failing ventricles remains poorly understood. Within the normal heart, Gα_i2_ is the dominant subtype, whereas Gα_o_ is about half the abundance of Gα_i2_ [78,79,80,81]. The role played by Gα_i2_ in pathological cardiac remodeling remains controversial [79,82,83,84], and little is known about the function of Gα_o_ in the pathophysiology of heart failure. We found that Gα_o_ is transcriptionally regulated by NRSF [10]. Furthermore, genetic reduction of Gα_o_ by mating with *Gnao1* knockout mice prevents the progression of pathological cardiac remodeling and cardiac dysfunction in dnNRSF-Tg mice and NRSF cKO mice (Figure 1). Similarly, in two other models of heart failure, mice with chronic pressure overload induced by transverse aortic constriction [85] and mice carrying a cardiac troponin T mutation [86], both show attenuated heart failure progression under conditions where Gα_o_ is genetically reduced. We also demonstrated that increased expression of Gα_o_ in the heart causes cardiac dysfunction. Mechanistically, Gα_o_ increases surface sarcolemmal L-type Ca^2+^ channel activity in ventricular cardiomyocytes, which, in turn, activates pathological Ca^2+^ signaling such as CaMKII activation, thereby impairing SR function and leading to pathological cardiac remodeling [10]. Single-cell RNA sequence data from human ventricular myocytes, which are publicly available, show higher expression of *Gnao1* and other NRSF target genes, including *NPPA*, *ACTA1* and *CACNA1H*, in dilated cardiomyopathy patients than in healthy control subjects [87]. These findings demonstrate that the NRSF-*GNAO1* transcriptional pathway may broadly underlie the development of human heart failure, implying that selective inhibition of Gα_o_ could be a novel and effective strategy for heart failure.

## 6. Epigenetic Regulators Associated with NRSF

**Figure 2:**
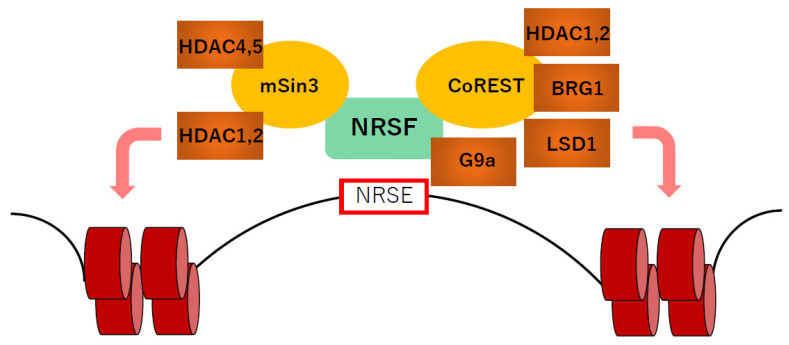
NRSF forms a repressor complex with epigenetic modifiers and cooperatively regulates gene expression. HDAC, histone deacetylase; LSD1, lysine (K)-specific demethylase 1A; NRSE, neuron-restrictive silencer element; NRSF, neuron-restrictive silencer factor.

A number of studies performed in non-cardiac cells have revealed that NRSF forms a complex with corepressors to repress transcription (Figure 2). NRSF has two repressor domains at the N- and C-terminal ends, respectively. The N-terminal repressor domain interacts with the mSin3A and mSin3B complexes that associate with histone deacetylases (HDAC) [7,88,89]. On the other hand, the C-terminal repressor domain interacts with the corepressor CoREST, which forms a complex with HDACs, the histone demethylase LSD1 and the ATP-dependent chromatin remodeling enzyme BRG1 [88]. The C-terminal repressor domain of NRSF also interacts with the histone methylase G9a independently of CoREST [90]. HDACs remove acetyl groups from histone tails, resulting in transcriptional repression by chromatin compaction [91]. LSD1 demethylates mono- and dimethylated H3 lysines K4 and K9, thereby repressing gene expression [92]. G9a monomethylates and dimethylates histone H3K9 (H3K9me1 and H3K9me2) and, to a lesser extent, H3K27 [93]. In cardiomyocytes, NRSF forms a complex with Class I HDACs and the Class IIa HDACs, HDAC4 and HDAC5, and suppresses *NPPA* and *NPPB* expression by modifying histone acetylation [7,89]. Class I HDACs are relatively ubiquitously expressed, while Class IIa HDACs, including HDAC4, HDAC5, HDAC7 and HDAC9, are expressed in a tissue-specific manner, most abundantly in heart, brain and skeletal muscle. In the heart, Class IIa HDACs have been reported to act as a signal-responsive suppressor of cardiac hypertrophy [94]. Phosphorylation at two conserved sites by Ca^2+^/calmodulin-dependent kinase and/or protein kinase D induced by hypertrophic stimuli causes nuclear export of Class IIa HDACs, resulting in the attenuation of NRSF-mediated repression of the fetal gene program during cardiac remodeling [89]. These findings suggest that NRSF needs association with epigenetic machinery at least in part for regulation of cardiac gene expression.

The actions of HDACs have been studied in a variety of heart failure models. In a mouse model of chronic pressure overload induced by transverse aortic constriction, treatment with a pan-HDAC inhibitor (HDACi) blunted the hypertrophic response and slowed the progression toward heart failure [95,96]. Cardiac fibrotic markers were also reduced in the ventricle, and HDACi application reduced collagen production in isolated cardiac fibroblasts [96]. HDACi treatment also ameliorated established hypertrophy [95]. Similar cardioprotective effects were produced through cardiomyocyte-specific deletion of Class I HDACs (HDAC1 and HDAC2) [97,98]. On the other hand, cardiomyocyte-specific deletions of Class II HDACs promoted hypertrophic responses [99]. In addition, administration of a clinical-stage HDACi (givinostat) improved cardiac function in two diastolic dysfunction models; the Dahl salt-sensitive hypertensive rat and normotensive diastolic dysfunction mice induced by aging [100]. The HDACi-mediated improvement seen with these models appears to be unrelated to blood pressure, cardiac hypertrophy, changes in the isoform of expressed cardiac sarcomeric proteins, or regulation of gene transcription. Instead, the improvement correlates with enhanced relaxation of myocardial fibers mediated by direct deacetylation of sarcomeric proteins [100]. In a recent study using a feline model of diastolic dysfunction and heart failure induced by pressure overload, another HDACi (vorinostat) had a similar effect on relaxation properties of isolated myocardial fibers [101]. Moreover, vorinostat ameliorated cardiac hypertrophy and cardiac fibrosis in a manner similar to that seen in mouse models of pressure-overload-induced hypertrophy [96,101]. Suppression of HDACs appears to reduce cardiac hypertrophy, protect against oxidative damage, reduce inflammation, inhibit fibrosis and modulate the composition extracellular matrix [97,102]. However, taking into consideration that cardiac-specific deletion of HDAC1 and HDAC2 results in neonatal death, with arrhythmia and dilated cardiomyopathy [91], more precise studies will be needed before establishment of a clinical application.

The role of LSD1 has also been studied in various heart failure models. LSD1 deletion in cardiac myofibroblasts attenuates the remodeling induced by transverse aortic constriction, whereas LSD1 deletion in cardiomyocytes triggers mild cardiac hypertrophy and dysfunction [103]. Knockdown of LSD1 prevents cardiac fibroblast activation by inhibiting the TGFβ pathway, as evidenced by downregulation of Ang-II-induced TGFβ1 expression and Smad2/3, p38, ERK and JNK phosphorylation. On the other hand, LSD1 deletion from cardiomyocytes downregulated CoREST and NRSF accompanied with reactivation of ANP and BNP. LSD1 seems to maintain the integrity of the CoREST complex, so that deletion of LSD1 causes downregulation of CoREST protein [104]. CoREST reduction triggers a decrease in the level of LSD1 and de-repression of NRSF-responsive gene expression [105]. How LSD1 deletion downregulates NRSF in cardiomyocytes and how LSD1 affects the TGFβ pathway in cardiac fibroblasts remain unknown, however.

G9a forms a heteromeric complex with GLP (G9a-like protein, also known as EHMT1 or KMT1D), another enzyme catalyzing the methylation at H3K9 in vivo [106]. Genetic analysis showed that, in vivo, the histone methyl-transferase activity of G9a is more important than that of GLP and that neither can compensate for the loss of the activity of the other [107]. G9a and GLP are essential for normal development, and KO mice deficient in either gene die in utero (at E9.5) due to severe growth defects [106]. H3K9me2 accumulates during cardiac development and keeps adult cardiomyocytes locked into their terminally differentiated state in which the fetal gene program is suppressed. However, pathological hypertrophic stimuli increase expression of miR-217, which downregulates G9a/GLP and leads to a reduction in H3K9me2. This decrease in H3K9me2 causes cardiomyocytes to be in a less differentiated state and de-represses the fetal gene program [108]. It remains unclear whether the effects of G9a described above are mediated by NRSF, however.

All these studies demonstrate that alterations in histone acetylation and/or histone H3 lysine methylation play an important role in the regulation of cardiac genes mediated by NRSF [109]. We anticipate that further studies into how each component of the epigenetic regulators contributes to the regulation of the cardiac gene program via NRSF and cardiac homeostasis will lead to a better understanding of the molecular mechanisms underlying the pathological cardiac remodeling and heart failure.
